# A Critical Assessment of the Effects of Bt Transgenic Plants on Parasitoids

**DOI:** 10.1371/journal.pone.0002284

**Published:** 2008-05-28

**Authors:** Mao Chen, Jian-Zhou Zhao, Hilda L. Collins, Elizabeth D. Earle, Jun Cao, Anthony M. Shelton

**Affiliations:** 1 Department of Entomology, Cornell University/New York State Agricultural Experiment Station (NYSAES), Geneva, New York, United States of America; 2 Department of Plant Breeding and Genetics, Cornell University, Ithaca, New York, United States of America; University of California Davis, United States of America

## Abstract

The ecological safety of transgenic insecticidal plants expressing crystal proteins (Cry toxins) from the bacterium *Bacillus thuringiensis* (Bt) continues to be debated. Much of the debate has focused on nontarget organisms, especially predators and parasitoids that help control populations of pest insects in many crops. Although many studies have been conducted on predators, few reports have examined parasitoids but some of them have reported negative impacts. None of the previous reports were able to clearly characterize the cause of the negative impact. In order to provide a critical assessment, we used a novel paradigm consisting of a strain of the insect pest, *Plutella xylostella* (herbivore), resistant to Cry1C and allowed it to feed on Bt plants and then become parasitized by *Diadegma insulare,* an important endoparasitoid of *P. xylostella.* Our results indicated that the parasitoid was exposed to a biologically active form of the Cy1C protein while in the host but was not harmed by such exposure. Parallel studies conducted with several commonly used insecticides indicated they significantly reduced parasitism rates on strains of *P. xylostella* resistant to these insecticides. These results provide the first clear evidence of the lack of hazard to a parasitoid by a Bt plant, compared to traditional insecticides, and describe a test to rigorously evaluate the risks Bt plants pose to predators and parasitoids.

## Introduction

Development and commercialization of corn and cotton varieties expressing insecticidal proteins (Cry toxins) from *Bacillus thuringiensis* (Bt corn and Bt cotton) have offered an alternative to traditional synthetic insecticides for control of important agricultural pests. Bt corn and Bt cotton have been adopted by farmers in 22 countries to control lepidopteran pests such as corn borers (mainly *Ostrinia nubilalis*) in corn and the budworm-bollworm complex (*Heliothis virescens, Helicoverpa spp., Pectinophora gossypiella*) in cotton [1]. Recent reports indicate the use of Bt crops has resulted in economic benefits to growers and reduced the use of conventional insecticides [2]; however, their potential impact on predators and parasitoids remain a concern [3]. The effects of Cry toxins on predators have been recently reviewed by Romeis et al. [4]. Predators usually feed on several different species of insects during their lifetime, thus exposing themselves to several potential sources of the toxin. The situation with parasitoids is fundamentally different since they generally complete their development within a single host. Thus, if their host feeds on Bt plants it is likely that the parasitoid will become exposed to the Cry toxin. Negative impacts of Bt toxins on non-target parasitoids have been reported in some plant-herbivore-parasitoid (tritrophic) studies with Bt plants and susceptible herbivores that were fed Bt plant tissue [5]–[9]. Although all these negative impacts on parasitoids could be indirect (i.e. mediated by poor host-quality) [4], the direct effects (toxicity) of Bt could not be ruled out. To test this rigorously it is necessary to have an insect pest that is resistant to the toxin but, despite the widespread area grown to Bt corn and Bt cotton (42.1 million ha in 2007) [10], no corn or cotton insect has evolved sufficiently high resistance to plant-expressed Bt proteins that it can readily survive on Bt corn or cotton. Thus, the direct and indirect effects of Cry toxins on the parasitoids cannot be clarified in these cropping systems.

The diamondback moth, *Plutella xylostella*, is the world's most destructive insect pest of cruciferous plants, which are widely grown as vegetables (e.g. cabbage, broccoli, and cauliflower) and field crops (e.g. canola), and the estimated annual cost for controlling this insect is $US 1 billion [11]. In the past 50 years, *P. xylostella* has become one of the most difficult insects to manage, primarily because of resistance evolution in some parts of the world to every class of insecticide used extensively against it [12]–[14]. It is also the first insect reported to have evolved resistance to a Cry toxin [15]–[16] due to foliar sprays of Bt products (no Bt crucifers have been commercialized). Further laboratory selection of a field-collected Cry1C-resistant population has resulted in *P. xylostella* with Cry1C resistance levels exceeding 60,000-fold [17]. This highly resistant strain can readily survive on broccoli plants expressing Cry1C [17], and thus provides a unique system to test the toxicity of Cry1C not only to *P. xylostella* but also to its parasitoids. *Diadegma insulare* is the most important parasitoid of *P. xylostella* in North America [18]–[20] while its close relative, *D. semiclausum*, is the dominant parasitoid in most other parts of the world [11]. Adults from both species prefer to deposit their eggs in the second larval stage of *P. xylostella*. The emerging parasitoid larva completes its development within the *P. xylostella* larva, then emerges from the last larval stage and pupates. The *P. xylostella* larva dies and the adult *D. insulare* emerges from its pupa and searches for a host.

The objective of this study was to study the direct and indirect effect on *D. insulare* of the Cry1C protein, when expressed in a plant or presented as a purified protein or in a commercial formulation. Cry1C was chosen since, like most Cry1 toxins, it is toxic to a broad range of Lepidoptera [21]. In order to eliminate host-quality mediated impacts on this non-target parasitoid, we used a Cry1C-resistant *P. xylostella* strain as the host of *D. insulare* and studied the potential for acute and indirect toxicity of Cry1C to *D. insulare*. Furthermore, to provide a proper comparison for risk assessment evaluations, similar bioassays were conducted with conventional insecticides widely used against *P. xylostella* using strains of *P. xylostella* that had evolved resistance to them. In this study, we first verified the high levels of resistance in strains of *P. xylostella* to the Cry1C protein, presented in Cry1C-expressing plants or as a purified toxin or in a formulated insecticide, and to four commonly used insecticides against *P. xylostella*: spinosad, λ-cyhalothrin, cypermethrin and indoxacarb. Then we assessed parasitism by *D. insulare* in populations of *P. xylostella* that were resistant or susceptible to Cry1C or the insecticides, and also the direct effects of these materials on *D. insulare*.

## Results

### Impact of Bt Plants, Cry1C Toxin and Formulated Insecticides on Different *P. xylostella* Strains

Bioassays confirmed the high levels of resistance of the *P. xylostella* strains to their respective insecticides (supporting information, Table S1). The Pearl strain had a resistance ratio (resistance ratio (RR)  = LC_50_ of tested strain/LC_50_ of the susceptible strain G88) of 8,445 to spinosad, while the Waipio strain showed a high level of resistance to indoxacarb (RR = 321), λ-cyhalothrin (RR = 4,700) and cypermethrin (RR = 373). The Cry1C-resistant strain had RR values of 1,436 to the formulated Cry1C product (MC) and >4,167 to purified 1C.

We confirmed that most individuals of the resistant *P. xylostella* strains could complete development when reared on a diet of broccoli leaf treated with the appropriate insecticide or Cry1C source. In addition, the Cry1C-resistant strain of *P. xylostella* was able to complete development when reared on Cry1C broccoli leaves (supporting information, Figure S1). These two sets of results demonstrated that we had the appropriate strains of *P. xylostella* to use for testing the effects of Bt plants, Cry1C, and formulated insecticides on the parasitism rates of *D. insulare.*

### Bioactivity of Cry1C after Ingestion by *P. xylostella* Larvae

To confirm that *D. insulare* was exposed to active Cry1C toxin when it developed inside Cry1C-resistant *P. xylostella* larvae, we fed Cry1C-resistant *P. xylostella* larvae Cry1C in one of three forms: purified Cry1C toxin, a product containing Cry1C (MC) or a Cry1C producing plant. After ingesting Cry1C, the Cry1C-resistant larvae were ground up and put into a solution, and the solution was applied to cabbage leaf disks that were fed to Cry1C-susceptible larvae. All extracts from Cry1C-fed *P. xylostella* larvae were toxic to susceptible *P. xylostella* (*F* = 69.113, *df* = 9,30; *P*<0.001) (Table 1). This suggests that the endoparasitoid *D. insulare* was exposed to active Cry1C toxin during its lifetime in its host when *P. xylostella* larvae fed on Cry1C. Cry1C from the bodies of Cry1C-resistant *P. xylostella* that fed on Bt plants caused the highest mortality of Cry1C-susceptible *P. xylostella* (67.50%±6.29), followed by the MC liquid formulation (52.50%±4.79), and the purified Cry1C (37.50%±4.79).

**Table 1:** Toxicity of Cry1C residue to Cry1C-susceptible *Plutella xylostella* larvae after the toxin had been consumed by Cry1C-resistant *P. xylostella* larvae

Treatment	Mortality % (Means ±SE)
Cry1C-R on extract from Cry1C-R larvae reared on MC (Cry1C)-containing diet	52.50±4.79 B
Cry1C-R on extract from Cry1C-R larvae reared on purified Cry1C-containing diet	37.50±4.79 C
Cry1C-R on extract from Cry1C-R larvae reared on Cry1C broccoli	67.50±6.29 A
Cry1C-R on control diet	0 D
Cry1C-R on normal broccoli leaf	2.50±2.50 D
Cry1C toxin extraction buffer	0 D
MC (Cry1C) washing buffer	0 D
Purified Cry1C washing buffer	0 D
Cry1C broccoli washing buffer	0 D
Control	2.50±2.50 D

A total 40 susceptible *P. xylostella* larvae was used in each treatment with 4 replicates (10 larvae/replicate). Means (±SE) followed by different letters are significantly different based on Fisher's LSD mean separation test (*P*<0.05)

### Parasitism of *D. insulare* on Different Untreated *P. xylostella* Strains

**Figure 1:**
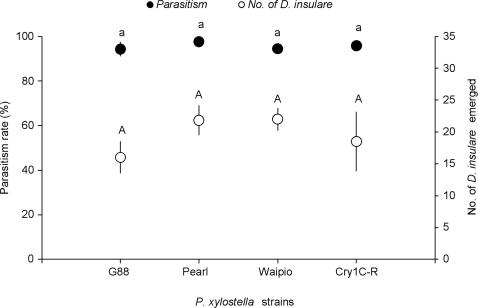
Parasitism rate (%) and the number of *Diadegma insulare* emerged from different untreated *Plutella xylostella* strains. Means (±SE) marked with different lower-case letters or capital letters are significantly different based on Fisher's LSD mean separation test (*P*<0.05).

The parasitism rates of *D. insulare* did not significantly differ among different *P. xylostella* strains when they were not challenged by a toxin (*F* = 0.746, *df* = 3,16; *P* = 0.541) (Fig. 1). More than 90% of *P. xylostella* larvae from each treatment were parasitized by *D. insulare* and the total number of *D. insulare* which emerged from different *P. xylostella* strains after being parasitized was not significantly affected (*F* = 1.088, *df* = 3,16; *P* = 0.382) (Fig. 1), confirming that the different genetic backgrounds of the *P. xylostella* strains did not discernibly alter *D. insulare* parasitization success.

### Parasitism of *D. insulare* on *P. xylostella* was Reduced by Formulated Insecticides but not by Cry1C Toxin

**Figure 2:**
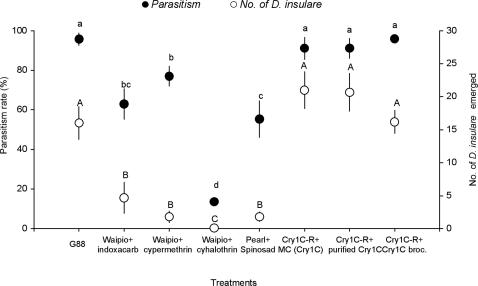
Parasitism rate (%) and the number of *Diadegma insulare* emerged from different *Plutella xylostella* strains treated with formulated insecticide, Bt toxin or left untreated. Means (±SE) marked with different lower-case letters or capital letters are significantly different based on Fisher's LSD mean separation test (*P*<0.05).

Thirty *P. xylostella* larvae (second instar) were fed with insecticide-treated or Cry1C toxin-treated broccoli leaves or Cry1C-expressing broccoli leaves for 24 h, before exposure to one pair of *D. insulare* in each replicate (5 replicates for each treatment). The total number of *D. insulare* individuals that emerged from parasitized *P. xylostella* larvae was significantly affected by different treatments (*F* = 29.876, *df* = 7,51; *P*<0.001) (Fig. 2). The number of *D. insulare* that emerged from the control group (16.00±2.45) was not significantly different from the number (16.12±1.72) that emerged from the Cry1C-resistant larvae reared on Cry1C-expressing broccoli. Similarly, parasitism rates of *D. insulare* on treated insecticide-resistant *P. xylostella* larvae were significantly lower than on a non-treated control or Cry protein-treated resistant larvae (*F* = 21.887, *df* = 7,51; *P*<0.001) (Fig. 2). For example, parasitism was reduced to 13.6%, the lowest in this study, when the Waipio strain was treated with 50 mg(AI)/L λ-cyhalothrin. By contrast, the parasitism rate of *D. insulare* on Cry1C-resistant *P. xylostella* reared on Cry1C-expressing broccoli was not significantly different from that of the control (susceptible) G88 strain of *P. xylostella*.

### Contact Toxicity of Bt Plants, Cry1C or Formulated Insecticides to *D. insulare*

Contact toxicities of different treatments on *D. insulare* female and male adults were pooled, because no significant difference was found between female and male adults in each treatment. Contact toxicities of different treatments on *D. insulare* were significantly different (Table 2; *χ^2^* = 80.55, *df* = 7, *P*<0.001). No *D. insulare* individuals died in vials treated with the purified Cry1C toxin or Cry1C broccoli plant for 2, 12, 24, 48 and 72 h, respectively, and this was not significantly different from the control group. All tested insecticides were very toxic to *D. insulare*, in comparison with purified Cry1C and the Cry1C plant. All *D. insulare* adults died after contacting residues of λ-cyhalothrin for 2 h, cypermethrin and spinosad for 12 h, and indoxacarb for 24 h.

**Table 2:** Survival (%) of the parasitoid *Diadegma insulare* after contact with different formulated insecticides, Cry1C toxin or Cry1C broccoli plants for different time periods

Treatment	% Survival after different inoculation times (h)					Significant difference
	2	12	24	48	72	
Indoxacarb	80	15	0	-	-	B
Cypermethrin	20	0	-	-	-	C
λ-cyhalothrin	0	-	-	-	-	D
Spinosad	15	0	-	-	-	C
MC (Cry1C)	100	100	100	100	100	A
Purified Cry1C	100	100	100	100	100	A
Cry1C broccoli	100	100	100	100	100	A
Control	100	100	100	100	100	A

Survivals of *D. insulare* in different treatments followed by different letters are significantly different (survival analysis with the Kaplan-Meier procedure and Logrank Test, *P*<0.05)

## Discussion

One of the perceived risks associated with growing Bt plants is their potential to adversely affect non-target organisms, including biological control agents such as parasitoids and predators. Although many field studies to date have shown negligible impact on non-target organisms in comparison with conventional insecticides [4], [22], [23], negative effects of Bt toxins on parasitoids have been reported with Bt plants both in laboratory [5]–[8] and field studies [24], as well as in field studies with frequent Bt sprays [25]. Unlike predators, which are often generalists and feed on several different prey species, parasitoids usually complete their development on a single host individual and are likely to be adversely affected if their Bt susceptible hosts are treated with Bt toxin and are weakened or killed [26], a phenomenon usually referred to as host-quality mediated effects. However, in a study in which sub-lethal (to the host) concentrations of Cry1Ac were added to an artificial diet, Walker et al. [27] found that the poor-quality hosts that developed on the diet had a greater effect on a parasitoid than the Cry1Ac toxin. Similarly, Vojtech et al. [9] assumed that the negative impact of Bt corn on the parasitoid *Cotesia marginiventris* was host-quality mediated when it developed inside susceptible *Spodoptera littoralis* larvae that had fed on transgenic Bt corn.

In the present study, a Cry1C-resistant *P. xylostella* strain that easily survived on Cry1C-expressing plants was used as the host of *D. insulare.* These Bt plants did not negatively affect the parasitism rate or emergence of *D. insulare*, while conventional formulated insecticides significantly reduced both (Fig. 2). However, it should be noted that the parasitism rates in some insecticide treatments were relatively high in comparison with the very low number of *D. insulare* emerged (Fig. 2). This might be due to a lack of an acute effect of the insecticide residue in the host on *D. insulare* larvae thereby allowing *D. insulare* larvae to destroy the host, although *D. insulare* did not successfully develop to adult stage. While further study is needed to verify this hypothesis, we suggest that the number of parasitoids emerged is a more important measure of success since the parasitoids may suppress the next generation of the pest. Our study is the first to make these direct comparisons and to confirm that the negative impact of Bt plants on parasitoids is due to the poor host quality as the result of ingestion and susceptibility to Bt toxin, rather than direct toxicity to the parasitoid. Generally, lepidopteran-active Bt toxins are considered to lack direct toxicity to parasitoids and predators [28], but it is difficult to confirm this hypothesis. Direct feeding and choice experiments for adult parasitoids, including *Cotesia plutellae* [29], [30], revealed no harm to the parasitoid. However, these studies did not determine whether the parasitoids were actually exposed to the biologically active toxin. In contrast, direct toxicity of conventional insecticides to some parasitoids, either through contact or ingestion, has been frequently reported [31]–[33]. In our study, all parasitoids were killed within 24 h in our contact bioassays with λ-cyhalothrin, cypermethrin, spinosad, and indoxacarb, while none died from the Cry1C toxin or Cry1C broccoli plants (Table 2). Similarly, in contact bioassays Xu et al. [32] found λ-cyhalothrin, indoxacarb, and spinosad had dramatic negative effects on *D. insulare*, while Bt-insecticides had none.

Schuler et al. [30] used a population of *P. xylostella* resistant to Cry1Ac and examined the indirect effect on *C. plutellae*. They found that when *C. plutellae* parasitized Cry1Ac-resistant *P. xylostella* that had fed on Cry1Ac-expressing oilseed rape, the parasitoid did not suffer any deleterious effects. While their findings are important and suggest that Cry1Ac was not toxic to *C. plutellae*, several questions remained. First, they did not confirm that the parasitoid was exposed to a biologically active form of the toxin. Our studies clearly demonstrated that Cry1C in the resistant host was in an active form because the residue was toxic to Cry1C-susceptible *P. xylostella* (Table 1). This provides strong evidence that the Cry toxin was not toxic to the parasitoid. Second, they did not make direct comparisons with purified or formulated Cry1Ac, nor with other commonly used insecticides for control of *P. xylostella*. Such comparisons are essential for a risk benefit analysis and regulatory decisions.

Using Bt broccoli and resistant strains of *P. xylostella*, we have demonstrated that the Cry1C toxin expressed in plants had no deleterious effects on an important parasitoid of *P. xylostella*, compared to commonly-used formulated insecticides. This suggests the safety to parasitoids for the current Bt corn and cotton crops that are expressing Cry1 toxins. Furthermore, it provides valuable information to regulatory authorities as Bt crucifer crops are being developed for commercial use [34]. However, if a new toxin with a different insecticidal mechanism is expressed, additional studies on its effect on a parasitoid may be required.

Parasitoids may influence the evolution of resistance in their host populations. Populations of parasitoids will be disadvantaged when the commonly used formulated insecticides we tested (λ-cyhalothrin, cypermethrin,indoxacarb, and spinosad) are applied in the field compared to application or transgenic expression of the lepidopteran-active Cry toxins expressed in plants. Decreased parasitoid populations would likely allow higher pest populations to develop and increase selection for resistance. Evolved resistance to Bt plants remains a major concern and it is possible that parasitoids may play a useful role in diminishing the likelihood of evolution of Bt resistance [35].

The widespread use of Bt plants for insect pest management has led to many ecological and regulatory questions about their effects on non-target organisms. We suggest that future studies examining the potential effects of Bt plants on non-target organisms utilize Bt-resistant insects or non-susceptible insects, whenever possible, to eliminate any spurious effects due to host quality. Furthermore, for a proper risk benefit assessment of pest management alternatives, we suggest that comparisons to commonly-used formulated insecticides be included in such tests.

## Materials and Methods

### Insects

Four strains of *P. xylostella* were used: 1) Cry1C-resistant strain (Cry1C-R) which can easily survive on Cry1C Bt broccoli plants [17], [36], [37]; 2) Pearl strain which has a high level of resistance to spinosad [38]; 3) Waipio strain which is resistant to indoxacarb [14], λ-cyhalothrin and cypermethrin and; 4) Geneva 88 strain [12], [16] which is susceptible to Cry1C and conventional insecticides. The hymenopteran endoparasitoid, *Diadegma insulare*, was originally field collected in Florida and subsequently reared in our greenhouse according the procedures of Xu and Shelton [39].

### Bt Plants, Cry1C and Formulated Insecticides

Transgenic broccoli (*Brassica oleracea* L., var. ‘*italica’* “Green Comet”) plants producing a high level of Cry1C (1.09–1.12 μg/g fresh leaf tissue) were used in this study [40], [41]. The expression of the Cry1C in the broccoli plants was verified when plants were 4–5 weeks old by screening them with *P. xylostella* neonates [42]. MC, a liquid formulation of Cry1C (active ingredient (AI): 15% w/w) protoxin expressed in and encapsulated by transgenic *Pseudomonas fluorescens* (Mycogen, San Diego, CA), was used to test the impact on *D. insulare*, in comparison with purified Cry1C toxin produced in *Escherichia coli*.

Four formulated insecticides, commonly used against *P. xylostella*, were tested: spinosad (SpinTor 2 SC, Dow AgroSciences, Indianapolis, IN), indoxacarb (Avaunt 30% WDG, DuPont Crop Protection, Newark, DE), λ-cyhalothrin (Warrior T 1CS, Zeneca Ag, Wilmington DE) and cypermethrin (Ammo 2.5 EC, FMC Co., Philadelphia, PA).

### Impact of Bt Plants, Cry1C Toxin and Formulated Insecticides on Different *P. xylostella* Strains

In order to confirm resistance in the strains noted above and to determine a concentration of each insecticide or Cry1C toxin (MC and purified Cry1C) that killed the susceptible *P. xylostella* strain yet had no significant impact on the development of resistant *P. xylostella* strains that would be parasitized by *D. insulare,* cabbage leaf dip bioassays [12], [16], [17] were used for the *P. xylostella* Pearl, Waipio and G88 strains. Each assay included 5–6 concentrations plus a control, using 5 leaf disks for each concentration. Ten second instars (0.2–0.3 mg/larva) were placed on each of the leaf disks inside 30-ml plastic cups and mortality was checked after 72 h at 27±1°C. Preliminary tests showed that the Cry1C-R strain was very tolerant to MC and the purified Cry1C toxin. The Cry1C-R strain was tested with 1000 mg (AI)/L of the purified Cry1C powder. Based on the results of the formal bioassays, 50 mg (AI)/L each of indoxacarb, λ-cyhalothrin and cypermethrin solutions were used for the Waipio strain and 100 mg (AI)/L of spinosad solution was used for the Pearl strain to evaluate the impact of insecticides on *D. insulare* within the *P. xylostella* larvae (the selected concentration killed all *P. xylostella* larvae from the susceptible strain but very few from the resistant strains).

In addition, mortalities of the different *P. xylostella* strains, after being treated with insecticides or Cry1C broccoli or Cry1C toxins, were investigated to correct the parasitism rates of *D. insulare* on different treated *P. xylostella* strains. This was done because the selected concentration of each insecticide, Cry1C toxins or Cry1C broccoli still killed a few *P. xylostella*, although each *P. xylostella* resistant strain was considered resistant to each formulated insecticide, Cry1C toxin or the Bt plant. Five replicates, each with five second instar *P. xylostella* from the Pearl strain, were fed with 100 mg (AI)/L of spinosad treated broccoli leaves for 2 d (exposure time to insecticide or Bt plant was the same in the following experiments), then transferred to normal (non-Bt) broccoli leaves and reared to adult emergence in a rearing chamber. Mortality of *P. xylostella* was recorded by correcting the parasitism rate of *D. insulare* on spinosad-treated *P. xylostella* from the Pearl strain. Mortalities of the Waipio strain (after treatment with 50 mg (AI)/L of indoxacarb, λ-cyhalothrin or cypermethrin) and Cry1C-R strain (feeding on 1000 mg (AI)/L MC and purified Cry1C treated broccoli leaves or Cry1C broccoli leaves for 2 d) were investigated as described above for the Pearl strain.

### Bioactivity of Cry1C After Ingestion by *P. xylostella* Larvae

In order to examine whether the Cry1C toxin (from MC, purified Cry1C, or Bt plants) was still biologically active after being consumed by Cry1C-resistant *P. xylostella*, *P. xylostella* second instars from the Cry1C-resistant strain were fed with MC or a purified Cry1C solution applied to the surface of an artificial diet, or Cry1C broccoli leaves. Before Cry1C-resistant larvae were infested, diet in each cup was covered with 2 ml of 1000 mg (AI)/L of MC or purified Cry1C with 0.1% Bond spreader sticker and left for 2 h at room temperature. A 0.1% Bond spreader sticker solution-treated diet and non-Bt broccoli leaf were used as controls. After Cry1C-R larvae fed on the diet or the Cry1C broccoli leaves for 2 d, they were collected into different 1.5 ml Eppendorf vials. The larvae from three treatment groups (MC, purified Cry1C and Cry1C broccoli) were washed with distilled water 4 times before being ground with a pestle in 20 μl Cry1C toxin extraction Buffer (supplied in Cry1C ELISA kit, EnviroLogix Inc., Catalog No. AP 007, Portland, Me.) per mg of larvae. The biological activity of Cry1C residues from the Cry1C-resistant insects was determined by evaluating the residues using leaf dip bioassays on Cry1C-susceptible *P. xylostella*, as described above. Ten second instars from the susceptible G88 strain were placed on each of the leaf disks inside 30-ml plastic cups with 4 replicates. Mortality was checked after 72 h at 27±1°C.

### Parasitism of *D. insulare* on Different Untreated *P. xylostella* Strains

Thirty second instar *P. xylostella* of each strain (G88, Cry1C-R, Pearl and Waipio) were placed on individual broccoli leaves in 125-ml flasks of water. Each flask was placed in a wood-framed cage with netted sides (50×50×50 cm) in the rearing chamber. One pair of *D. insulare* (within 2 d after emergence) was released into the cage after being supplied with 10% sugar water and allowed to mate for 24 h. Each treatment was replicated 5 times. The *P. xylostella* larvae were taken from the cage after 24 h and reared until *P. xylostella* adult or *D. insulare* emergence. The number of *P. xylostella* and *D. insulare* emerged from each treatment was recorded. Parasitism rate (%) was calculated as: (1-number of *P. xylostella* emerged/number of *P. xylostella* inoculated)×100.

### Parasitism of *D. insulare* on Different *P. xylostella* Strains Exposed to a Bt Plant, Cry1C Toxin or Formulated Insecticides

Second instar *P. xylostella* were fed with insecticide-treated or Cry1C toxin-treated broccoli leaves or Cry1C broccoli leaves for 24 h, before exposure to *D. insulare*. The concentration of indoxacarb, λ-cyhalothrin or cypermethrin solutions used for leaf treatments was 50 mg (AI)/L with 0.1% Bond spreader sticker; the concentration of the spinosad solution was 100 mg (AI)/L; the concentration of the MC or purified Cry1C solution was 1000 mg (AI)/L. After *P. xylostella* second instars fed on the treated broccoli leaf for 24 h, they were transferred to another broccoli leaf treated with a formulated insecticide, the Cry1C toxin or a Cry1C broccoli plant set in a 125-ml flask of water and exposed to *D. insulare* as described above with the same experimental designs and conditions. *Plutella xylostella* larvae in each treatment were taken from the cage after being exposed to *D. insulare* for 24 h, then transferred to normal broccoli leaves and reared in the chamber. The number of *P. xylostella* and *D. insulare* emerging in each treatment was recorded. Parasitism rate was calculated as above and corrected with *P. xylostella* mortality (from second instars to adults after *P. xylostella* larvae were treated with formulated insecticides or Cry1C) according to Abbott's formula [43]. The non-treated G88 strain was used as a control to compare the impact of different formulated insecticides and Cry1C on *D. insulare*.

### Contact Toxicity of Bt Plants, Cry1C or Formulated Insecticides to *D. insulare*

The same concentration of each insecticide used above (50 mg (AI)/L for indoxacarb, λ-cyhalothrin and cypermethrin; 100 mg (AI)/L for spinosad) was used for evaluating contact toxicity to *D. insulare* adults, compared with the Cry1C toxin (1000 mg (AI)/L) and a Cry1C broccoli plant. Glass vials (length 9.5 cm, dia. 2.5 cm) were filled with different formulated insecticide solutions with 0.1% Bond spreader sticker, then drained and left at room temperature for 2 h on a paper towel to dry, leaving an insecticide or Bt toxin residue on the inner wall of the glass vial. A Cry1C toxin residue was also made as described above using 1000 mg/L purified Cry1C toxin (>800×higher than the expression level in Cry1C broccoli) to simulate the impact of Bt sprays in the field on parasitoids. Cry1C broccoli leaves were placed inside each glass vial to evaluate the contact toxicity of the Bt plant on *D. insulare*. One pair of *D. insulare* (within 2 d after emergence) was released into each vial. Each treatment was replicated 10 times with one pair of *D. insulare* for each replicate. Mortality of male and female *D. insulare* was recorded at 2, 12, 24, 48 and 72 h for each treatment after inoculation.

### Statistical Analyses

The POLO program was used for probit analysis of efficacy of insecticide or Cry1C (dose-response) data [44]. Differences in efficacy were considered significant when 95% FL (fiducial limits) of LC_50_ values did not overlap. The resistance ratio (RR) was calculated by dividing the LC_50_ of the tested strain by the corresponding LC_50_ of the susceptible G88 strain. Data on mortality of *P. xylostella*, parasitism rate, total number of *D. insulare* emerged were analyzed by the GLM (generalized linear model) program and Fisher's protected LSD mean separation test (SPSS version 11.5 for windows, 2002). All percentage data were arcsine square root transformed, as necessary, before univariate analysis. Survival analyses of *D. insulare* adults after contact with synthetic insecticide or Cry1C toxin residue were conducted using the Kaplan-Meier procedure and Logrank Test [45].

## Supporting Information

Table S1 (0.03 MB DOC)

Figure S1 (0.08 MB DOC)
