# Supplementary material for: A Critical Assessment of the Effects of Bt Transgenic Plants on Parasitoids
Source: PLoS One. 2008 May 28;3(5):e2284. doi: 10.1371/journal.pone.0002284 (PMC2409141; doi:10.1371/journal.pone.0002284)
Supplement: Table S1 — (0.03 MB DOC) [file pone.0002284.s001.doc]

**Table S1. Efficacy of different insecticides on *Plutella xylostella*** second instars from different strains after 72 h at 27 ºC

| Insecticide | *P. xylostella* strain | n | Slope (±SE) | LC_50_, (95%FL),  mg (AI)/L | χ2 (df) | RR^a^ |
| --- | --- | --- | --- | --- | --- | --- |
| Indoxacarb | G88 | 250 | 1.39 (0.19) | 0.27  (0.17-0.39) | 0.78 (3) | 1 |
|  | Waipio | 250 | 2.65 (0.39) | 86.60  (45.10-125.00) | 3.46 (3) | 321 |
| Cypermethrin | G88 | 300 | 1.19 (0.20) | 0.44  (0.14-0.98) | 6.26 (4) | 1 |
|  | Waipio | 300 | 0.96 (0.17) | 164.00  (59.00-368.00) | 4.59 (4) | 373 |
| λ-cyhalothrin | G88 | 250 | 0.75 (0.12) | 0.03 | 10.9(3) | 1 |
|  | Waipio | 300 | 0.72 (0.11) | 141.00  (80.00-266.00) | 1.36 (4) | 4700 |
| Spinosad | G88 | 250 | 1.40 (0.19) | 0.03  (0.01-0.05) | 3.32 (3) | 1 |
|  | Pearl | 250 | 2.22 (0.40) | 253.35  (176.36-332.95) | 1.95 (3) | 8445 |
| MC (Cry1C) | G88 | 300 | 1.98 (0.20) | 1.73 (0.71-5.03) | 10.91 (3) | 1 |
|  | Cry1C-R | 300 | 2.10 (0.42) | 2485.00  (1225.24-3706.63) | 3.94 (3) | 1436 |
| Purified Cry1C | G88 | 300 | 1.90 (0.20) | 0.24 (0.19-0.30) | 2.96 (4) | 1 |
|  | Cry1C-R | 50 | (30.00±5.50% mortality at 1000 mg (AI)/L) | | | >4167 |

^a^RR, resistance ratio= LC_50_ of tested strain / LC_50_ of the susceptible strain G88
