# Supplementary material for: A Critical Assessment of the Effects of Bt Transgenic Plants on Parasitoids
Source: PLoS One. 2008 May 28;3(5):e2284. doi: 10.1371/journal.pone.0002284 (PMC2409141; doi:10.1371/journal.pone.0002284)
Supplement: Figure S1 — (0.08 MB DOC) [file pone.0002284.s002.doc]

**Selecting the proper dose for testing the resistant *P. xylostella* strains.** After *P. xylostella* larvae from the Cry1C-R strain were allowed to feed on Cry1C broccoli, Pearl strain larvae

were treated with 100 mg (AI)/L spinosad and Waipio strain larvae were treated with 50 mg (AI)/L indoxacarb, λ-cyhalothrin and cypermethrin solutions for 2 d, respectively, less then 28% of the *P. xylostella* died in each treatment group confirming that most individuals of the resistant *P. xylostella* strains could successfully finish development after being treated with the appropriate formulated insecticides or Bt plant (*F*=2.927, *df*=7,32, *P*=0.17). These doses were used in subsequent tests

**Fig. S1. Cumulative mortality of different strains of *Plutella xylostella* larvae from second instar to adult after being treated with formulated insecticides, Cry1C toxin or Cry1C broccoli plants for 2 days**

Column (means ± SE) marked with different lower-case letters are significantly different based on Fisher’s LSD mean separation test (*P*<0.05)
